# Transgene Biocontainment Strategies for Molecular Farming

**DOI:** 10.3389/fpls.2020.00210

**Published:** 2020-03-03

**Authors:** Michael Clark, Maciej Maselko

**Affiliations:** ^1^Applied Biosciences, Macquarie University, North Ryde, NSW, Australia; ^2^CSIRO Health and Biosecurity, Canberra, ACT, Australia; ^3^CSIRO Synthetic Biology Future Science Platform, Brisbane, QLD, Australia

**Keywords:** biocontainment, molecular farming, pharmaceuticals, plant synthetic biology, metabolic engineering, transgene, industrial enzymes, biofuel

## Abstract

Advances in plant synthetic biology promise to introduce novel agricultural products in the near future. ‘Molecular farms’ will include crops engineered to produce medications, vaccines, biofuels, industrial enzymes, and other high value compounds. These crops have the potential to reduce costs while dramatically increasing scales of synthesis and provide new economic opportunities to farmers. Current transgenic crops may be considered safe given their long-standing use, however, some applications of molecular farming may pose risks to human health and the environment. Unwanted gene flow from engineered crops could potentially contaminate the food supply, and affect wildlife. There is also potential for unwanted gene flow into engineered crops which may alter their ability to produce compounds of interest. Here, we briefly discuss the applications of molecular farming and explore the various genetic and physical methods that can be used for transgene biocontainment. As yet, no technology can be applied to all crop species, such that a combination of approaches may be necessary. Effective biocontainment is needed to enable large scale molecular farming.

## Molecular Farming

The potential of engineered plants as low-input production platforms for large-scale production of pharmaceuticals is an area of active research. Examples of plant made pharmaceuticals (PMPs) with global markets include human insulin, human serum albumin (HSA) and HIV-neutralizing antibodies. There is a large need for human insulin due to the high incidence of diabetes world-wide, which includes a substantial undersupplied market in Asia. Plant production of insulin could meet this shortfall at a price diabetics in this region could afford (Stoger et al., 2014). Over 500 tons per year of HSA are necessary to treat fetal erythroblastosis, fluid loss due to burn injuries, hypoproteinemia, and ascites caused by cirrhosis of the liver (Chen et al., 2013). InVitria, a division of Ventria Bioscience has developed Optibumin, a rice-derived HSA that has already been commercialized (He et al., 2011)^1^. An estimated 5 tons of HIV-neutralizing antibody is needed to supply 10 million women with the minimal amount necessary to prevent HIV (Shattock and Moore, 2003). Two publicly funded projects, Pharma-Planta and Future-Pharma have produced HIV-neutralizing antibodies in tobacco and corn seeds for clinical trials. It is hoped their production platform could be used in affected areas to produce microbicides “in the region for the region” (Stoger et al., 2014). For all three examples, there is a greater demand for these products than there is a supply of them, and this is particularly the case in under-developed countries. The immense scalability of molecular farming could meet the demand at a price that matches the economic situation of the target areas.

Molecular farming also has the potential to enhance the production of pharmaceuticals naturally produced in plants such as the anti-cancer drug Taxol (paclitaxel) and artemisinin, a crucial anti-malarial compound. The plants that synthesize these compounds do so in low concentrations and grow slowly resulting in only minute quantities of the desired compound (Buyel, 2018). Taxol was originally extracted from the Pacific yew, *Taxus brevifolia*, where the bark from a single 100 year old tree yields about 300 mg of Taxol, enough for only one dose (Horwitz, 1994). Today Taxol is produced by Bristol-Myers Squibb using a semi-synthetic process starting with Taxol intermediates extracted from the Yew tree needles. Using the needles rather than the bark is non-destructive but the Yew tree is still slow growing and the intermediates require expensive purification (Howat et al., 2014). Similarly low amounts of artemisinin – 0.01–1.4% dry weight (DW) – accumulate in sweet wormwood, *Artemisia annua* (Ikram and Simonsen, 2017). Engineering the biosynthetic pathways for these compounds into heterologous plants optimized for molecular farming could boost supplies and reduce costs (Wurtzel et al., 2019).

Although the complete biosynthetic pathway for the production of Taxol hasn’t been elucidated the biosynthetic pathway to produce the first committed product, taxadiene, has been engineered into *Nicotiana benthamiana*. The full biosynthetic pathway for artemisinin has also been engineered into *N. tabacum*. Both tobacco species are production platforms for molecular farming due to their fast growth and high biomass production. The most successful attempt to biosynthetically produce artemisinin took two large sections of the metabolic pathway for artemisinin and genetically engineered them separately into three different cellular compartments (chloroplast, nucleus, and mitochondria). The resulting heterologous expression of artemisinin at ∼0.8 mg/g DW was less than in the native plant, which can reach 31.4 mg/g DW (Zhang et al., 2009; Malhotra et al., 2016). This can in part be explained by the complexity of the gene expression and regulation of the biosynthetic pathway (Ikram and Simonsen, 2017). A mg/g comparison also doesn’t reflect *N. tabacum’s* faster growth and higher biomass production when compared to *A. annua*. The genetic engineering of *N. benthamiana* to express a taxadiene synthase gene, which produces taxadiene from geranylgeranyl diphosphate (GGPP), produced 11–27 μg/g DW taxadiene. Further suppression of the phytoene synthase gene and addition of methyl jasmonate increased taxadiene accumulation to 35 μg/g DW (Hasan et al., 2014). The successful *de novo* production of taxadiene could lead to the development of a heterologous plant system that biosynthesises Taxol. Future improvements in metabolic engineering could see a breakthrough in how these high value compounds are produced.

Using plants for the production of enzymes or other proteins impacts both the safety and the potential activity of the isolated products. Plant production is also free from human pathogens – a major concern in mammalian cell culture production systems – and free from endotoxins, which are a risk in bacterial systems (Commandeur et al., 2002). Protein glycosylation patterns can be manipulated in plants, including to produce ‘humanized’ glycosylation patterns (Hanania et al., 2017; Mercx et al., 2017). This is important for complex glycoproteins such as monoclonal antibodies or membrane proteins as glycosylation can affect protein stability, subcellular targeting, biological activity, and immunogenicity (He et al., 2014). The glycosylation of asparagine or arginine side-chains is similar for plants and mammals until the glycan reaches the Golgi apparatus. In plants the side-chain can be modified by the attachment of an α(1,3)-linked fucose or β(1,2)-linked xylose, whereas in mammals there can be the attachment of an α(1,6)-linked fucose, β(1,4)-linked galactose or sialic acid (Gomord et al., 2010). In some cases, plant glycosylation produces proteins with higher pharmacological activity than proteins produced by bacterial or mammalian cells. For example, plant production systems produce taliglucerase alfa, a mannose-terminated glycoprotein for the treatment of Gaucher’s disease, where terminal mannose residues are needed to bind to macrophage mannose receptors. In contrast, mammalian cell system production requires post-production glycosylation modifications to expose terminal α-mannose residues (Grabowski et al., 2014). There is, however, the possibility alternative glycosylation will increase the chance of immunogenicity. Several plant production systems have been engineered to give the recombinant protein human glycosylation patterns (Kallolimath et al., 2016).

Plants can also produce large volumes of industrial compounds. Examples of plant made industrial compounds (PMIs) include cellulases and amylases for bioethanol production, xylanases to enhance animal feed and oxidation/reduction enzymes such as laccases and peroxidases for paper manufacturing (Van Der Maarel et al., 2002; Bailey et al., 2004; Clough et al., 2006; Shen et al., 2012; Hood and Requesens, 2014). Currently, bioethanol is produced by using starch derived from corn. To enhance this process Syngenta’s genetically modified (GM) corn, Enogen, expresses an α-amylase enzyme, which catalyzes the breakdown of starch into glucose (Que et al., 2014). Corn is also used as animal feed or human food, meaning that there is competition for agricultural space. Plant biotechnology could enable utilizing more of the cellulose and hemicellulose to be used to produce biofuels. The US company Agrivida increased ethanol production by 55% by engineering corn to express cell wall degrading enzymes *in planta* (Zhang et al., 2011).

Transgenic plants have been developed to be a source of fibrous animal proteins such as collagen, keratin, silk, and elastin (Börnke and Broer, 2010). The Israeli biotechnology company CollPlant developed a tobacco line to produce recombinant human collagen (Stein et al., 2009). Typically, medical collagen comes from animal or human cadavers which pose an infection risk from prions (Pammer et al., 1998). Additionally, the extraction process forms unwanted inter- and intra-molecular bonds, which reduce the solubility and the ability of the collagen to form into more desirable highly structured scaffolds (Zeugolis et al., 2008). Whereas, the plant-derived collagen is cross-link and pathogen free, so it can be modified for the desired application.

For maximal scalability and cheap production molecular farming is conducted with field grown crops. A good case study is the plant biotechnology company Infinite Enzyme, which uses field grown corn to heterologously produce 1.5 million kg of cellulase annually (the amount needed for a 190 million liter per year cellulosic biofuel facility). To produce and process field grown corn only $2 million in capital investment – for dry milling and defatting equipment – was required; with $11.7 million per year in operating costs ($7.8/kg enzyme). In contrast, a microbial fermentation system, which requires tanks and the associated infrastructure, would require $100 million in upfront capital investment. A further $15 million per year would also be needed in operating costs ($10/kg enzyme)^2^. However, the economic advantages of using a field grown crop, must be balanced out by the possibility of the transgene contaminating other crop production.

## The Troubled History of Transgene Escape

While molecular farming has the potential to lower the cost of medications and industrially useful compounds, the growth of these technologies is contingent on the containment of the transgenes. Challenges of transgene biocontainment are not just hypothetical; there are two salient examples of the need for effective containment – the ProdiGene and StarLink affairs (Murphy, 2007). ProdiGene produced a transgenic corn that expressed a vaccine for preventing bacteria-induced diarrhea in pigs, and while the vaccine protein was non-toxic to humans, strict exclusion from the human food chain was required (Hileman, 2003). StarLink’s corn crop was genetically engineered with a gene for resistance to the herbicide glufosinate, and it contained a variant of the pest control *Bacillus thuringiensis* (Bt) protein (Cry9C) – it also lacked approval for food use. In 2000, StarLink’s transgenic corn contaminated millions of tons of non-transgenic corn throughout the United States. Government officials have said StarLink’s developer, Aventis CropScience, failed to ensure farmers kept StarLink corn separate from other varieties^3^. The contaminated corn was recalled for disposal, costing Aventis an estimated $500 million (Murphy, 2007). In 2002, ProdiGene failed to eradicate plants that had seeded from their previous season’s transgenic corn crop. This led to the contamination of non-transgenic soybeans. ProdiGene’s failure to manage their transgenic corn crop resulted in 12,000 tons of soybean being destroyed. The combined cost to ProdiGene was about $3.5 million with an additional US government fine of $250,000 (Thayer, 2002).

The fallout from the ProdiGene and StarLink affairs was lasting. In response the molecular farming industry pushed for tighter regulations regarding the approval process for molecular farming crops (Murphy, 2007). In 2003, the Animal and Plant Health Inspection Service (APHIS) of the US Department of Agriculture (USDA) introduced the requirement that crops engineered to produce PMIs be grown under permit. Previously, a GM PMI producing crop could be cultivated under notification, which expedited the permitting procedure (Federal Register [FR], 2005). A full discussion of the interplay between regulation and molecular farming is beyond the scope of this review. Although, it is worth making the point that regulatory hurdles remain a barrier to molecular farming. For example, Syngenta’s development of Enogen cost several 100 million dollars, a lot of which was due to it taking almost 6 years to pass USDA’s regulatory review process (Wang and Ma, 2012). It is promising though that in 2011 Enogen met USDA’s requirements to be fully deregulated. In doing so Enogen became the first plant genetically engineered for industry to be granted this status (Wang and Ma, 2012). The success of Enogen shows a pathway to the commercialization of a PMI production platform.

Inefficient transgene biocontainment has impacted international trade. Japan and South Korea halted imports of corn from the United States during the StarLink corn incident. Exports of wheat to Japan and South Korea were also briefly stopped in 2013, after a GM wheat event MON71800 – developed by Monsanto to be glyphosate-tolerant, was found growing in a field. Monsanto paid $2.1 million to farmers to compensate the loss of export income and reputational damage, and paid $250,000 to several wheat growers’ associations^4^. In 2016, a sister event (the same DNA was inserted into a different genomic location) – MON71700 – was found to have contaminated a field in the state of Washington. The 22 plants descend from a field trial conducted by Monsanto from 1998 to 2001^5^. In both cases the reoccurrence of the GM wheat was unexplained. The precedent of a GM crop re-emerging more than a decade after a trial stokes public concern over food safety and biosecurity. Such concerns will continue to impact the adoption and development of plant biotechnologies (Murphy, 2007). In order to foster acceptance of transgenic plant production systems there must be proper containment and security at all levels of production.

## Importance of Biocontainment

There are concerns from the public and from within the scientific community that molecular farming could threaten non-GM agriculture, the environment, and human health. Without adequate biocontainment, neighboring non-GM crops or weeds could receive transgenes and transgenic seeds could contaminate seed storage (Mallory-Smith and Sanchez Olguin, 2011; Gressel, 2015). Contamination worries many in the food industry, who are not involved with molecular farming, but could suffer financially and in terms of public confidence if theirs or any other major edible crop became contaminated (Murphy, 2007). Contamination can impact international trade between countries that have legal restrictions on importing transgenic products (Lu, 2003). There are also environmental concerns stemming from the possibility of crop-to-wild transgene flow. In most cases, the few resulting offspring from crop × wild crosses will be outcompeted due to being less locally adapted than the wild type (Gressel, 2015) although the transfer of herbicide resistance genes to weeds, including invasive species, could increase the difficulty of eradicating them. It is improbable, but a transgene could also spread from an engineered crop to a weed and then from that weed to another crop. In this way, weeds that contain the transgene could act as a reservoir for that transgene allowing spread to non-GM crops.

In some cases, molecular farming could potentially pose a risk of humans or animals being harmed through inadvertent exposure to an unsafe level of recombinant protein (Breyer et al., 2012). The majority of PMPs currently in production, such as antibodies, growth hormone, insulin and most other proteins, are expected to have no pharmacological effect when ingested (Goldstein and Thomas, 2004). Instead the gastro-intestinal tract will degrade most PMPs to harmless peptides or amino acids. However, many exceptions may exist in the future, and some plant pharmaceuticals, such as oral vaccines, are designed to be active when ingested. There is also potential for skin or eye contact and inhalation of the recombinant protein as well as the potential allergenicity of the plant itself (Breyer et al., 2012). The human health threats are heightened by the fact that a plant product could enter the human food or animal feed chain. An event that is more likely if the transgenic crop is also a food crop, as was seen for ProdiGene.

As well as potentially exposing humans or animals to a harmful compound, contamination can affect the quality of related crops. The North American Miller’s Association were concerned that the transgene for amylase expression in Enogen could spread into other corn varieties and result in lower quality tortillas, corn puffs, and bread (Waltz, 2011). The advance of agriculture will likely see new crop varieties generating novel products such as cotton engineered to be red in color. In order to maintain the phenotypic integrity of transgenic and non-transgenic cultivars effective biocontainment will be required.

The potential economic, environmental, and health threats from molecular farming can be greatly reduced through controlling the flow of the transgene. It’s also important to point out that the level of threat from transgene escape depends on the nature of the contamination. Trace mixing of seed that contains a toxic protein is unlikely to be harmful due to dilution. However, the introgression of a transgene, which expresses a toxic protein, into a neighboring crop or weed could seriously contaminate human food or animal feed chains. Although any contamination, regardless of risk, will likely impact public support for GM agriculture.

## Transgene Containment Technologies

**FIGURE 1:**
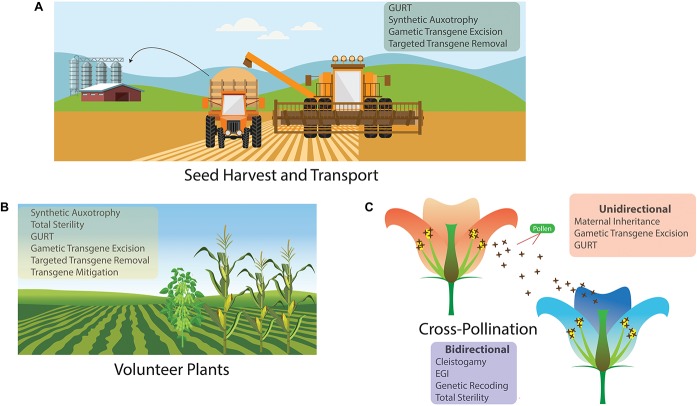
The three main pathways for unwanted contamination or gene flow in an agricultural setting; with a list of the genetic biocontainment technologies that could be used to reduce the possibility of the gene flow occurring. **(A)** Seed dispersal during harvest and transport. **(B)** Contamination from volunteer plants. **(C)** Genetic biocontainment can limit pollen-mediated gene flow unidirectionally, where transgenes are prevented from spreading from the transgenic crop into neighboring plants, and it can operate bidirectionally, where gene flow into the transgenic crop is also limited.

Gene flow is a process where the frequency of a gene changes in a population and can occur through gametes, an organism or groups of organisms moving from one population to another. The potential for there to be gene flow into or from a crop depends on the crop’s pollination strategy, on the size of the crop, seed size and viability, and whether there are compatible species within pollination distance (Mallory-Smith and Sanchez Olguin, 2011). Figure 1 details the three main ways that transgenes can spread into the environment. Volunteer plants – plants that have self-seeded from a previous season’s crop – can contaminate the next season’s crop if they are accidentally harvested alongside the intended crop (Michael et al., 2010). Transgenes may also spread in seeds that can be spilled during the harvest and transfer of seed. Lastly, cross-pollination can lead to either transgenes escaping into neighboring plants or introgression from neighboring plants into the transgenic crop (Gressel, 2015). As we are primarily concerned with the movement of genes into another population, pollen transfer is the form of gene flow that is of most concern.

There are essentially two approaches for minimizing gene flow: containment and mitigation. Containment aims to stop the flow of the gene from the crop and mitigation focuses on preventing the gene from establishing in a significant proportion of the population (Gressel, 2015). Containment can be physical or biological. Physical containment provides a barrier, such as a greenhouse, filters in the lab or isolation distances in the field. There are also efforts to conduct molecular farming underground, e.g., in unused mines, which provide an even higher degree of physical containment^6^. So far there are no documented cases of physical containment failing in the laboratory or greenhouse (Gressel, 2015). Whereas, the shortcomings of geographic isolation were shown when transgenes from GM glyphosate-resistant creeping bentgrass, *Agrostis stolonifera*, were found in non-agronomic bentgrass up to 3.8 km beyond the control area perimeter (Reichman et al., 2006). With the unreliability of geographic isolation in many situations it is preferable to avoid the use of crop plants grown for human or animal consumption.

Alternative plant production platforms have been developed to reduce the risk of contamination. Some examples of non-food and non-feed crops include tobacco (*N. benthamiana*), duckweed (*Lemna minor*), microalgae (*Chlamydomonas reinhardtii*), and moss (*Physcomitrella patens*) (Yao et al., 2015). As can be seen from Table 1, tobacco and moss are popular production platforms. The use of these plants prevent introgression of a transgene into a plant used for food or feed. If a crop plant is to be used, crops that can be crossed with weedy relatives, such as the sunflower, *Helianthus annuus*, should be avoided.

**TABLE 1:** Examples of plant made pharmaceuticals.

Product	Disease	Plant production host	Clinical trial status	Company	References
HIV/HSV microbicide MB66	HIV/HSV	Tobacco	Phase I	Mapp Biopharmaceutical, United States	https://mappbio.com/product-development/
Zmapp^TM^	Ebola Zaire virus	Tobacco	Phase II/III	Mapp Biopharmaceutical, United States	https://mappbio.com/product-development/
VEN BETA	Gastroenteritis	Rice	Preclinical phase	Ventria Bioscience	https://ventria.com/
VEN120	Inflammatory bowel disease	Rice	Phase II	Ventria Bioscience	https://ventria.com/
Moss-aGal (agalsidase)	Fabry disease	Moss	Phase I	Greenovation Biopharmaceuticals	https://www.greenovation.com/home.html
Moss-FH	C3 Glomerulopathy	Moss	Preclinical phase	Greenovation Biopharmaceuticals	https://www.greenovation.com/home.html
H1N1 vaccine	Seasonal influenza	Tobacco	Phase III	Medicago, United States	https://www.medicago.com/en/pipeline/
H5N1 vaccine	Pandemic influenza	Tobacco	Phase II	Medicago, United States	https://www.medicago.com/en/pipeline/
Rotavirus vaccine	Rotavirus vaccine	Tobacco	Phase I	Medicago, United States	https://www.medicago.com/en/pipeline/
Optibumin	Loss of albumin	Rice	On Market	InVitria, United States	https://invitria.com/
Non-Hodgkin’s lymphoma vaccine	Non-Hodgkin’s lymphoma	Tobacco	Phase I	Icon Genetics, Germany	https://www.icongenetics.com/
Vibrio cholerae	Cholera	Potato	Phase I	Arizona State University	Tacket, 2005
Heat-labile toxin B subunit of *Escherichia coli*	Diarrhea	Potato	Phase I	Arizona State University	Tacket, 2005
Capsid protein Norwalk virus	Diarrhea	Potato, Tomato	Phase I	Arizona State University	Zhang et al., 2006
Antibody against hepatitis B	Vaccine purification	Tobacco	On market	CIGB, Cuba	Kaiser, 2008
ISOkine^TM^, BIOEFFECT^TM^ EGF Serum (human growth factors and cytokines)		Barley	On market	ORF genetics	https://orfgenetics.com/

Sound biocontainment and rapid production of recombinant protein can be achieved using a transient expression system which does not result in a transgene integrated in the germline. One method to establish a transient expression system is agroinfiltration where the bacteria *Agrobacterium tumefaciens –* acting as a vector for the gene of interest – is injected or vacuum infiltrated into leaf cells (Whaley et al., 2011). Another approach is to use plant RNA viruses (Yusibov et al., 2006). Both of these approaches can be combined, where agroinfiltration is used to deliver RNA viral vectors into the leaves of a plant. This process, called ‘magnifection’ combines the transfection efficiency of *A. tumefaciens*, the post-translational modifications of a plant and the high expression yield obtained with viral vectors (Marillonnet et al., 2005). In all of these approaches the transferred DNA is expressed but not integrated into the germline. The tobacco *N. benthamiana* is most often used as the production platform due to the ease with which it can be transformed. Compared to the time it takes to establish a stable transgenic plant line – 6 months to a year – transient expression systems can produce recombinant protein within 3–5 days (Yao et al., 2015). This is ideal for combating sudden viral epidemics, such as severe acute respiratory syndrome (SARS) or Ebola. Transient expression systems, as a consequence of not introducing transgenes into germline tissue, don’t risk contaminating food through transgene outflow into non-GM crops or their wild relatives (Huafang Lai and Jake Stahnke, 2013). However, *Agrobacterium* infiltration is labor intensive, which was a barrier to transient expression supplying sufficient supplies of an Ebola vaccine (Yao et al., 2015).

Whole-plant production platforms remain attractive due to their scalability but for some applications *in vitro* systems are preferable. Current *in vitro* technologies include plant–cell suspension and hairy root cultures. Plant–cell suspensions are typically derived from new tissue formed over a plant callus, which has been cultivated on solidified media. The clumps that easily break apart can be transferred to liquid media. If a homogenous culture forms, the fermentation of the plant cells can be conducted using similar techniques to fermenting lower eukaryotes (Fischer et al., 1999). Cell suspension cultures have sound containment and have a quick development cycle but are a much less scalable production platform, when compared to transgenic plants (Santos et al., 2016). Hairy root cultures are differentiated cultures of transformed roots generated by infection with *Agrobacterium rhizogenes* (Häkkinen et al., 2014). Hairy root culture can be grown with simple defined media like undifferentiated cells, but it has greater genetic stability and it is highly scalable. These features make it suitable for producing pharmaceutical proteins at an industrial-scale (Guillon et al., 2006). However, *in vitro* techniques require sophisticated and sterile laboratory settings. If the scalability and low-cost potential of plant production of PMPs or PMIs is to be realized, plants need to be grown in fields.

The higher contamination risk from growing plants in fields can be reduced by genetic containment which may exploit existing reproductive limitations or introduce them via genetic engineering. Many genetic approaches for containing plant transgenes have been investigated including cleistogamy, maternal inheritance, gametic transgene excision, synthetic auxotrophy, total sterility, and genetic use restriction technologies (GURT or Terminator). Other genetic containment technologies in development could be extended to plants, such as engineered genetic incompatibility (EGI), genetic recoding and targeted transgene removal (see Table 2). Many of these technologies work well for specific types of plants and can be enhanced by pairing them with other technologies.

**TABLE 2:** The important features of genetic biocontainment technologies.

	Weakened by introgression	Mitigates volunteer plants	Difficult to engineer	Transgene will persist	Unidirectional cross-pollination control	Bidirectional cross-pollination control	Demonstrated in plants
Cleistogamy				X		X	X
Gametic transgene excision		X			X		X
Synthetic auxotrophy	X	X	X				X
Total sterility		X				X	X
GURT		X			X		X
Maternal inheritance			X	X	X		X
EGI				X		X	
Genetic recoding	X		X	X		X	
Targeted transgene removal		X			X		

Cleistogamy, where there is self-pollination within a closed flower, is a promising tool to limit gene flow. Currently it suffers from some flowers opening, which allows for cross-pollination. Cleistogamy requires that the plant’s flower contain male and female parts and that there can be self-fertilization. Crops like rice have such flowers, but plants that have separate male and female flowers, like asparagus and spinach, or with unusual flower anatomies, such as corn, aren’t suited for cleistogamy. It was found that for imidazolinone herbicide resistant rice a few flowers opened enabling hybridization with weedy rice (Gealy, 2005). To combat this, rice was genetically engineered to enhance the percentage of cleistogamous flowers through incorporating the cleistogamous gene, ‘superwoman1.’ The engineered cultivar, in a variety of plots, had an outcrossing rate of 0.000% compared to the non-engineered cultivar, which ranged from 0.005 to 0.200% (Ohmori et al., 2012). The potential of cleistogamy is limited for GM food production as current practices tend to use higher-yielding hybrid rice varieties, which require parental lines that aren’t cleistogamous (Gressel, 2015). However, this would not be an issue for the molecular farming of high value compounds where cleistogamy could be used to restrict pollen mediated gene flow.

Synthetic auxotrophy works by genetically engineering a strain to depend on an externally supplied compound. The dependence can come from deleting essential genes that are needed, for example, to synthesize amino acids or co-factors that are necessary for crucial biological functions (Moe-Behrens et al., 2013). So far, this approach has found little traction for use in plants. There are isolated cases such as the duckweed *Lemna*, which has been engineered to be dependent on the addition of isoleucine through inactivating threonine deaminase expression (Nguyen et al., 2012). However, the genetic redundancy that is a common feature of plant genomes increases the difficulty in engineering recessive auxotrophic mutations. For most plants there are likely multiple proteins that catalyze the same reaction, which requires a large number of genetic changes to confer metabolic dependence (Last et al., 1991). The addition of potentially expensive chemicals, in itself a drawback, also requires changes to normal cultivation techniques. Synthetic auxotrophy can also fail due to introgression of genes from non-transgenic plants, which could restore the knocked out metabolic pathway.

Another approach exploits the maternal inheritance of plastids (e.g., chloroplasts). For the vast majority of higher plants, which display maternal inheritance, transgenes located in the plastid genome are unlikely to be transmitted to other plants by pollination (Maliga, 2004). Plastid engineering has therefore been employed to locate the transgene in the plastid genome, however, the advance of plastid engineering has been stymied by poor transformation protocols for plants other than tobacco. Transformation relies on many essential factors unique to the species and sometimes unique to the cultivar (Lu et al., 2013). There must be detailed knowledge of the plastid genome sequence including the regions in between genes suitable for transgene integration, there also needs to be an optimized DNA delivery system, as well as effective antibiotic selection and selectable marker genes. For several years the chloroplast genome sequences have been available for monocots, such as wheat and corn, but the chloroplast hasn’t been transformed due to the engineering complexity (Wani et al., 2015). This approach may also be less efficient than envisioned considering that species that were thought to strictly engage in maternal plastid inheritance still had about 0.4% plastid transmission via pollen (Avni and Edelman, 1991; Svab and Maliga, 2007). Additional problems are: proteins expressed in the chloroplast undergo different post-translational modifications, meaning that enzyme function might be altered (Grabsztunowicz et al., 2017); plastid transformation can also be laborious and time-consuming (Ruf et al., 2001).

Total sterility offers a sound basis for genetic biocontainment. Several crops are already sterile or have sterile varieties, such as cassava (*Manihot esculenta*), potatoes (*Solanum tuberosum*), and banana (*Musa acuminata*) (Celis et al., 2004; Heslop-Harrison and Schwarzacher, 2007; Sayre et al., 2011). As long as the sterility is not leaky, these crops would be safe candidates for molecular farming. A totally sterile plant can also be engineered by deleting genes that encode for gamete production (Kwit et al., 2011). The downsides are that total sterility requires plants to be vegetatively propagated by either tubers, tissue culture, cuttings, or artificial seed (Gressel, 2015). Total sterility could be used with tuber or bulb propagated crops, leafy vegetable crops and forestry. Whereas crops that are harvested for compounds accumulated in seeds would not be candidates for total sterility.

Gametic transgene excision uses a site-specific recombination system to excise a transgene. Currently, the efficiency of the recombinase is quite low, where 99% excision is considered to be high performing (Moon et al., 2011). This level of efficiency is too low to restrict transgene escape, however, it could be used to excise selectable marker genes used in the engineering of transgenic plants (Hu et al., 2013). Farmers are also not able to collect seed containing the transgene for future seasons unless the recombinase can be externally controlled, which alters normal cultivation practices (Ryffel, 2014; Gressel, 2015).

Genetic use restriction technologies were originally developed to prevent farmers from infringing on patents by saving seed. They have been some of the most controversial GM biotechnologies due to the widespread perception that they were designed to entrench a multinational corporation seed monopoly (Lombardo, 2014). GURTs use a tightly controlled genetic system to regulate the expression of a target gene. There are typically four components to this genetic system: the target gene, the target gene’s promoter, the trait switch and the genetic switch. The target gene needs to be activated by the promoter. In order to prevent leaky expression from unwanted promoter activity a blocker sequence separates the promoter from the target gene. The blocking sequence can in turn be removed through a cascade beginning with an external input, which will be amplified by the genetic switch. The amplified input becomes a biological signal that activates the trait switch. The trait switch usually encodes an enzyme, such as a site-specific recombinase that removes the blocker sequence (Lombardo, 2014). Without the blocker sequence there can then be transcription and expression of the target gene.

In part due to public opposition GURTs have never been commercialized. However, there is scope for GURTs to be used for biocontainment. For this, the GURT system would be linked to the transgene, so that when the GURT is activated there is expression of a disrupter gene that drives cell death. Disrupter genes typically encode for cytotoxins such as barnase and ribonuclease A (Mariani et al., 1990; Burgess et al., 2002; Gils et al., 2008; Zhang et al., 2012). There is no evidence that disrupter genes generate products that are toxic to humans or animals. However, it is possible that the potential health risk will add to the already controversial nature of using GURTs (Conner et al., 2003; Gressel, 2010). The other disadvantages to GURT are that it is a more expensive system, requiring exogenous inputs and there is greater difficulty in propagating a GURT crop.

## Future Biological Containment Technologies

The biocontainment technologies that have been developed in microbes could in some cases be extended to plants. Some of these technologies include, genetic recoding, targeted transgene removal and EGI.

Genetic recoding removes every instance of at least one codon for an amino acid in an organism’s genome and replaces it with another. The codon that has been removed can be replaced with a synonymous codon or it can then encode for a non-standard amino acid (NSAA) (Mukai et al., 2017). If an essential gene was recoded to require an amino acid not found in nature this would increase the stringency of an auxotrophy. Further, the genetic recoding could create reproductive isolation and block gene flow with non-recoded organisms due to incompatible genetic codes. *Escherichia coli* has been recoded so that the UAG stop codon instead incorporated a NSAA in the cores of essential enzymes. This conferred a dependence on synthetic metabolites for proper protein function, such that the bacteria were less capable of mutational escape and metabolic supplementation (Mandell et al., 2015). Following on from this the genetic recoding of plant genomes could confer better biocontainment. Despite the advance of the technology, we are unlikely to see recoding of higher organisms with ease in the near future due to the scale of changes needed in large genomes.

Another strategy could be to precisely remove the engineered genes instead of killing the whole organism. The spread of transgenes from volunteer plants or inadvertent seed dispersal could be mitigated by using a CRISPR-based system to selectively remove the transgene after the desired protein has been produced. In one such method, a genetically encoded device, termed DNAi, responds to a transcriptional input by degrading DNA adjacent to a synthetic CRISPR array. The DNAi system was shown to be non-toxic when carried in *E. coli*, and when activated it was able to reduce the number of viable cells by 1.9 × 10^–8^ making it one of the most effective switches for programmed cell death (Caliando and Voigt, 2015). This same mechanism could be engineered so that with the addition of a transcriptional input the transgene is degraded. An advantage of this system is that the removal of the transgene applies little selective pressure toward deactivating the genetic machinery; whereas directing whole organism death selects for mutations that lead to an organism’s survival.

The aforementioned biological containment technologies, with the exceptions of cleistogamy, genetic recoding, and total sterility, don’t prevent the flow of genes into the transgenic plant. This is an important consideration as unwanted gene flow can alter important traits in a genetically engineered organism. In order to restrict gene flow in both directions, plants could be engineered to be genetically incompatible with related plants such that the hybrid is less fit – this is known as underdominance. The model organism *Drosophila melanogaster* has been engineered such that engineered-WT hybrids display underdominance. This was achieved using a genetic construct to encode for two genes: the first encodes for a RNAi knockdown of the WT version of the gene *Rpl14*; the second gene is a refactored version of *Rpl4* such that it isn’t susceptible to RNAi knockdown. When the engineered organism was mated with WT flies there was a marked fitness reduction in the heterozygotes (Reeves et al., 2014). However, in order to be effective for biocontainment the underdominance must result in total sterility or death of the hybrids.

An artificial reproductive barrier, where the hybrids are non-viable, has been engineered in *Saccharomyces cerevisiae* using EGI. This system utilizes programmable transcriptional activators (PTAs) to overexpress a gene leading to lethality. Lethality in the engineered organism is avoided by editing the target sequence of the PTA, such that the PTA is unable to bind and overexpress the gene (Maselko et al., 2017). When there is a cross between the WT and the engineered organism, the PTA targets the WT PTA binding sequence and drives lethal levels of gene expression. Attempts have also been made at constructing a synthetic species of *D. melanogaster*, where an artificial reproductive barrier is engineered, however the goal of complete genetic isolation wasn’t achieved. The main difficulty proved to be getting strong activation of a lethal gene without the fitness costs associated with broad expression of the transactivating CRISPR machinery (Waters et al., 2018).

There is an inherent versatility to the use of PTAs, so that lethal overexpression of a target gene could theoretically be engineered in any sexually reproducing organism (Maselko et al., 2017). Proof of concept has so far only been established in *S. cerevisiae*. Although, it is conceivable that EGI could be extended to plants, where it could be used to generate many orthogonal strains of the same parent species which could each be used as production platforms for different compounds. If interbreeding can be prevented then the phenotypic integrity of transgenic cultivars could be protected. EGI could also be used to make synthetic auxotrophy more robust by preventing introgression from neighboring plants, which would otherwise compromise the auxotrophy.

## Transgene Mitigation Technologies

Even the most stringent containment system can fail. Technologies are therefore needed to reduce the chances of a transgene becoming established after escape. Transgenic mitigation involves linking the transgene to genes that confer a selective disadvantage. Weedy traits such as a propensity toward shattering, bolting, and greater height can be targeted (Gressel, 2015). Transgenic mitigation reduced the reproductive fitness of transgenic-weed oilseed rape hybrids. A dwarfing mitigator gene was linked to a herbicide resistance transgene, which reduced the reproductive fitness of the transgenic-weed hybrid to 0.9% of the competing weed’s reproductive fitness (Al-Ahmad and Gressel, 2006). However, there is the potential for the linkage of the mitigator gene to the transgene to be broken through meiotic crossing over. Additionally, there can be mutation of the mitigator gene so that it ceases to confer the deleterious phenotype. Both of these issues can in some part be addressed through linking another mitigator gene to the transgene, such that there are mitigating genes either side of the transgene (Gressel, 2015).

## Conclusion

Molecular farming has the potential to lower the cost of medication and industrial enzymes. However, in cases where the recombinant protein is potentially toxic, there are environmental and human health risks. The introgression of the transgene into a neighboring crop or weed may contaminate food or feed supplies. Any contamination event, such as in the high-profile cases of StarLink and ProdiGene, could jeopardize confidence in molecular farming. For these reasons there must be effective containment of transgenes.

There has been considerable progress in the development of biological containment technologies. For some species such as rice, cleistogamy could contain gene flow. For tubers and bulb propagated crops total sterility is practical. But for many species these technologies aren’t applicable. There is some promise that technologies like EGI combined with synthetic auxotrophy could contain gene flow. Further work in this area is needed to ensure the safety and widespread adoption of field grown molecular farming crops.

## Author Contributions

All authors listed have made a substantial, direct and intellectual contribution to the work, and approved it for publication.

## Conflict of Interest

MM is a co-founder and chief technical officer of NovoClade LLC.

The remaining author declares that the research was conducted in the absence of any commercial or financial relationships that could be construed as a potential conflict of interest.

## Footnotes

1 https://invitria.com/cell-culture-products/optibumin-lipid-reduced-recombinant-human-albumin-rhsa/

2 https://infiniteenzymes.com/technology-2/

3 https://web.archive.org/web/20070711190925/http://archives.cnn.com/2000/FOOD/news/10/18/conagra.grain.ap/

4 https://time.com/3582953/monsanto-wheat-farming-genetically-modified-settlement/

5 https://monsanto.com/company/media/statements/statement-gmo-wheat-plants/

6 https://www.wired.com/2004/05/drug-farms-forced-underground/
